# Deficiency in Poly(ADP-ribose) Polymerase-1 (PARP-1) Accelerates Aging and Spontaneous Carcinogenesis in Mice

**DOI:** 10.1155/2008/754190

**Published:** 2008-04-14

**Authors:** Tatiana S. Piskunova, Maria N. Yurova, Anton I. Ovsyannikov, Anna V. Semenchenko, Mark A. Zabezhinski, Irina G. Popovich, Zhao-Qi Wang, Vladimir N. Anisimov

**Affiliations:** ^1^Department of Carcinogenesis and Oncogerontology, N.N. Petrov Research Institute of Oncology, Pesochny-2, St. Petersburg 197758, Russia; ^2^Leibniz Institute for Age Research, Fritz Lipman e.V., 07745 Jena, Germany; ^3^Faculty of Biology and Pharmacy, Friedrich-Schiller-University of Jena, 07737 Jena, Germany

## Abstract

Genetic and biochemical studies have shown that PARP-1 and poly(ADP-ribosyl)ation play an important role in DNA repair, genomic stability, cell death, inflammation, telomere maintenance, and suppressing tumorigenesis, suggesting that the homeostasis of poly(ADP-ribosyl)ation and PARP-1 may also play an important role in aging. Here we show that PARP-1^−/−^ mice exhibit a reduction of life span and a significant increase of population aging rate. Analysis of noninvasive parameters, including body weight gain, body temperature, estrous function, behavior, and a number of biochemical indices suggests the acceleration of biological aging in PARP-1^−/−^ mice. The incidence of spontaneous tumors in both PARP-1^−/−^ and PARP-1^+/+^ groups is similar; however, malignant tumors including uterine tumors, lung adenocarcinomas and hepatocellular carcinomas, develop at a significantly higher frequency in PARP-1^−/−^ mice than PARP-1^+/+^ mice (72% and 49%, resp.; *P* < .05). In addition, spontaneous tumors appear earlier in PARP-1^−/−^ mice compared to the wild type group. Histopathological studies revealed a wide spectrum of tumors in uterus, ovaries, liver, lungs, mammary gland, soft tissues, and lymphoid organs in both groups of the mice. These results demonstrate that inactivation of DNA repair gene PARP-1 in mice leads to acceleration of aging, shortened life span, and increased spontaneous carcinogenesis.

## 1. Introduction

Poly(ADP-ribose) and poly(ADP-ribose) polymerases (PARPs) were discovered about 40 years ago, and have been shown to be important in many cellular processes, such as DNA replication, repair, recombination, cell proliferation and death, gene transcription, telomere maintenance, inflammation, as well as in carcinogenesis [1–6]. There are seventeen genes encoding PARP enzymes in mammalians, including PARP-1, PARP-2, PARP-3, and PARP-4/vPARP: PARP-5/Tankyrases-1 and PARP-6/Tankyrases-2 [7]. The best studied of these enzymes is PARP-1 that plays a primary role in the process of poly(ADP-ribosyl)ation. PARP-1 has been impicated in the pathogenesis of inflammatory and neurodegenerative disorders and cancer [5–9]. Upon DNA break induction by irradiation or alkylating agents, PARP-1 binds to DNA break sites and, by using NAD^+^, catalyzes poly(ADP-ribose) formation onto nuclear acceptor proteins [1, 2]. Superactivation of PARP-1 after extensive DNA damages can result in a damage of metabolic homeostasis due to an exhaustion of NAD^+^ substrate [10]. Previous studies have demonstrated that deficiency or inactivation of PARP-1 cause a high degree of genomic stability, characterized by aneuploidy, increased sister chromatid exchange, chromosome gain or loss, and telomere shortening [2, 11]. These data suggest that the defects in the homeostasis of poly(ADP-ribosyl)ation could accelerate both carcinogenesis [12] and aging [13]. Grube and Bürkle [14] first described a positive correlation between the poly(ADP-ribosyl)ation capacity of mononuclear blood cells with longevity of mammalian species. This correlation was explained in part by evolutionary sequence divergence [15]. In addition, there are correlation studies showing that PARP activity is decreased in aging human samples [16, 17]. Therefore, PARP-1 and poly(ADP-ribosyl)ation might act as a factor that limits the rate of aging [6]. However, whether deficiency of PARP-1 and poly(ADP-ribosyl)ation plays a causal role in aging lacks experimental evidence. In the present study, we set up a large cohort of mice lacking the major poly(ADP-ribose) synthesizing enzyme PAPR-1 (PARP-1^−/−^) to study life span, aging, and spontaneous tumorigenesis.

## 2. Material and Methods

### 2.1. Animals

The PARP-1 knock-out mice (PARP-1^−/−^) [18] and wild type mice (PARP-1^+/+^) were in 129/Sv background and maintained at the Department of Carcinogenesis and Oncogerontology, N. N. Petrov Research Institute of Oncology (St. Petersburg, Russia). Five to seven animals were kept in polypropylene cages (30 × 21 × 10 cm), respectively, under standard light/dark regimen (12 hours light: 12 hours darkness) at 22 ± 2°C and received standard laboratory chow [19] and tap water *ad libitum*. Animals were checked daily by animal care personnel and weekly by a veterinarian. The study was carried out in accordance with the regulations for ensuring the humane treatment of animals under the approval of the Committee on Animal Research of the N. N. Petrov Research Institute of Oncology.

### 2.2. Experiments

Seventy three female PARP-1^−/−^ mice (group 1) and 120 PARP-1^+/+^ mice (group 2) were used for the study. Once a month, simultaneously with weighting, the amount of drinking water and consumed food were measured and the rate of consumed food (grams) per mouse was calculated. Terms of puberty were estimated based on the age of vagina opening. Once every 3 months, vaginal smears, taken daily for 2 weeks from the animals, were cytological examined to estimate the phases of their estrous function. In the same period, rectal body temperatures of the mice were measured with an electronic thermometer, TPEM-1 (KMIZ, Russia). The animals were observed until their natural death or were sacrificed when moribund. The date of each death was registered, and the mean life span, the age at which 90% of the animals died, and the maximum life span were recorded.

### 2.3. “Open Field” Test of Locomotor Activity

Animals of each group were placed one by one in a plastic chamber (30 × 21 × 10 cm), at the bottom of which 5 × 4 squares (5 × 5 cm each) were drawn. The mice were observed moving in an “open field” and the following behavioral parameters were estimated: (1) the number of crossed squares in the field (a square was considered crossed if the animal stepped over its border with at least 2 paws); (2) the number of vertical sets (when the animal rose to its hind paws); and (3) the duration of standing reaction. To exclude the possibility of smell-associated orientation reaction, the chamber floor was wiped with a wet cloth after each animal. The mice were tested at the age of 6, 9, 12, and 18 months in the daytime from 10 am to 5 pm.

### 2.4. Studying Muscular Strength and Physical Fatigability

The mice were suspended on a string stretched to an altitude of 70 cm, so that they would hang by the string clutching at it with their front paws. The time until the moment of fatigue and fall was registered in seconds. Within 20 minutes, the mice were suspended again and the time, during which they managed to hold on, was measured. Discrepancy between these two indices was regarded as a parameter of physical restoration.

### 2.5. Biochemical Analysis

At the age of 4 and 20 months, 10 female mice of each PARP-1^−/−^ and PARP-1^+/+^ genotype were sacrificed by decapitation after overnight starvation. Samples of serum were obtained and stored in the −20°C for subsequent analysis. Biochemical parameters in the serum were analyzed using Flexor Vitalab (Vital Scientific, the Netherlands) and Kone 20 (Thermo electron, Labsystems, Finland) analyzers.

### 2.6. Histopathological Examination

All the animals that died or that were sacrificed when moribund were autopsied. At autopsy, their skin and all internal organs were examined. Any tumors identified were classified according to the recommendation of the International Agency for Research on Cancer (IARC) as “fatal” (i.e., those that directly caused the death of animal) or as “incidental” (for the cases in which animal died of a different reasons). All tumors, as well as the tissues and organs with suspected tumor development, were excised and fixed in 10% neutral formalin. After the routine histological processing, the tissues were embedded in paraffin. Thin (5–7 *μ*m) histological sections were stained with haematoxylin and eosine and were microscopically examined in a coded fashion. Tumors were classified according to IARC recommendations [20].

### 2.7. Statistics

Experimental results were statistically processed by the methods of variation statistics with the use of STATGRAPH statistic program kit. The significance of the discrepancies was defined according to the Student-*t* criterion, Fischer's exact method, *χ* ^2^, nonparametric Wilcoxon-Mann-Whitney, and Friedman RM ANOVA on Ranks. Student-Newman-Keuls method was used for all pairwise multiple comparisons. Coefficients of correlation were estimated by using the Spearman method [21]. Differences in tumor incidence were evaluated by the Mantel-Hansel log-rank test.

Parameters of Gompertz model were estimated using maximum likelihood method, nonlinear optimization procedure [22], and customized code in “MATLAB”; confidence intervals for the parameters were obtained using the bootstrap method [23].

For experimental groups, the Cox's regression model [24] was used to estimate relative risk of death and tumor development under the treatment compared to the control groups: *h* (*t*, *z*) = *h*_0_ (*t*) exp (*zβ*), where *h* (*t*, *z*) and *h*_0_ (*t*) denote the conditional hazard and baseline hazard rates, respectively, *β* is the unknown parameter for treatment group, and *z* takes values 0 and 1, being an indicator variable for two samples—the control and treatment group.

Semiparametric model of heterogeneous mortality [25] was used to estimate the influence of the treatment on frailty distribution and baseline hazard.

## 3. Results

### 3.1. Age-Related Body Weight Dynamics

**Figure 1:**
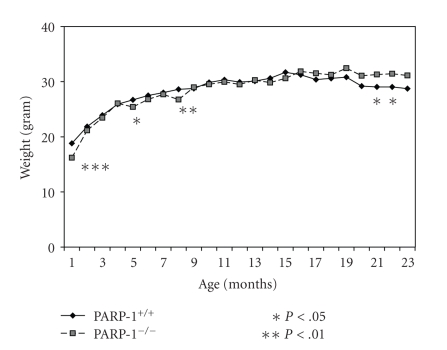
Age-related dynamics of body weight in PARP-1^−/−^ and PARP-1^+/+^ mice. PARP-1^−/−^ and PARP-1^+/+^ mice were measured every month for their body weight throughout a period of 23 months. Student's *t* test was performed to analyze the statistical significance of the results.

The mean body weight of mice increased with age both in the PARP-1^+/+^ and PARP-1^−/−^ groups, exceeding at 12 months the body weight of 3-month-old animals by 25% in the PARP-1^+/+^ group, and by 30% in the PARP-1^−/−^ group. Although there were no consistent differences in the mean body weight between the groups until the age of 21 months, in their later life, the mean body weight of PARP-1^−/−^ mice exceeded the body weight of the PARP-1^+/+^ mice (*P* < .05, Figure 1).

### 3.2. Age-Related Dynamics of Food Consumption

Regular measurement did not reveal any significant differences in the amount of food consumed between the PARP-1^+/+^ and PARP-1^−/−^ mice. It was practically the same during the all period of observation and varied from 2.5 ± 0.17 to 4.5 ± 0.91 g of food per mouse per day.

### 3.3. Age-Related Dynamics of Body Temperature

**Figure 2:**
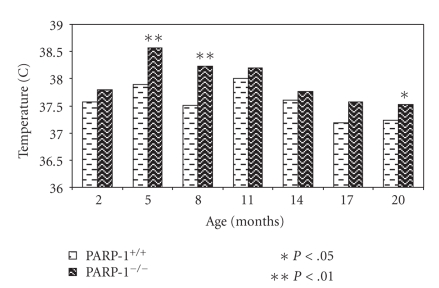
Age-related dynamics of body temperature in PARP-1^−/−^ and PARP-1^+/+^ mice. Bars represent the body temperature of PARP-1^−/−^ and PARP-1^+/+^ mice at indicated age. Student's *t* test was performed to analyze the statistical significance of the results.

An age-related decrease in body temperature was observed both in PARP-1^+/+^ and PARP-1^−/−^ mice. PARP-1^−/−^ mice had significantly higher body temperature than PARP-1^+/+^ mice at the age of 5, 8, and 20 months (Figure 2).

### 3.4. Age-Related Dynamics of Estrous Function

Puberty appeared earlier in PARP-1^−/−^ mice than in the PARP^+/+^ group. The age of vagina opening was 24 ± 0.2 days and 25 ± 0.3 days, respectively (*P* < .05). Investigations of the estrous function were repeatedly performed every 3 months, starting when the mice were 3 months of age. The following parameters of the estrous function were estimated: duration of the estrous cycle, the relative number of short (less than 5 days) and long (over 5 days) estrous cycles, as well as the relative number of animals with irregular cycles. Estrous cycle length was increased with age in both groups. However, the estrous cycle length was greatly increased in PARP-1^−/−^ mice, exceeding that of the level in PARP-1^+/+^ mice by 18% at the age of 17 months (Table 1). It is worthy noting that at the age of 20 months, the number of PARP-1^−/−^ mice with irregular cycles was significantly higher compared to PARP-1^+/+^ mice (72% and 30%, resp., *P* < .002). Thus, PAPR-1 deficiency resulted in the acceleration of age-related loss of the estrous function.

### 3.5. Age-Related Dynamics of Behavioral Parameters

**Figure 3:**
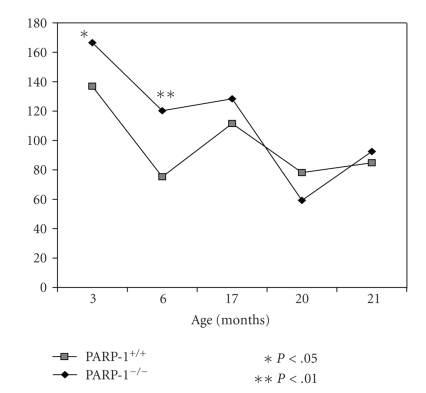
Age-related locomotor activity (“open field” test) in PARP-1^−/−^ and PARP-1^+/+^ mice. PARP-1^−/−^ and PARP-1^+/+^ mice were tested for their behavior by “open field” at the indicated age. The vertical axis represents the number of crossed squares at indicated age. Student's *t* test was performed to analyze the statistical significance of the results.

**Figure 4:**
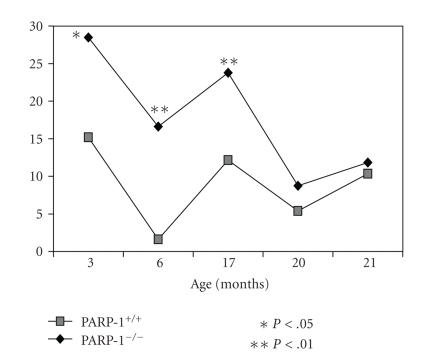
Age-related locomotor activity (“open field” test) in PARP-1^−/−^ and PARP-1^+/+^ mice. PARP-1^−/−^ and PARP-1^+/+^ mice were tested for their behavior by “open field” at the indicated age. The vertical axis represents the number of vertical sets at indicated age. Student's *t* test was performed to analyze the statistical significance of the results.

**Figure 5:**
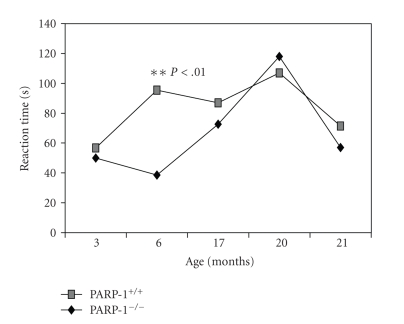
Age-related behavior (standing reaction duration) in “open field” test in PARP-1^−/−^ and PARP-1^+/+^ mice. The vertical axis represents the standing reaction duration at indicated age. Student's *t* test was performed to analyze the statistical significance of the results.

**Figure 6:**
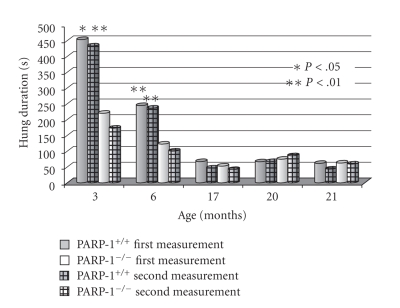
Age-related holding strength of PARP-1^−/−^ and PARP-1^+/+^ mice. The hung on the string duration of PARP-1^−/−^ and PARP-1^+/+^ mice was tested at the ages indicated by their holding a string, and the duration (in second) of holding a string was recorded. Student's *t* test was performed to analyze the statistical significance of the results.

During aging, a gradual decrease in locomotor activity in both parameters recorded (the number of crossed squares and the number of standing reaction) was observed in both groups whereas standing time, on the contrary, was increased (Figures 3–6). At the age of 3 and 6 months, PARP-1^−/−^ mice crossed squares 18% (*P* < .01) and 37% (*P* < .01) frequently than PARP-1^+/+^ mice, respectively. The number of the standing reaction that PARP-1 knock-out mice made exceeded that in controls: 47% at the age of 3 months (*P* < .05), 87% at 6 months (*P* < .01), and 49% at 17 months (*P* < .01). The duration of the standing reaction in 6-month-old knock-out mice was only 40% of the control. The time of clutching to a string (physical force parameter) was gradually reduced with the age in both groups (Figure 6). In the PARP-1^−/−^ mice, the duration of both the first and the second suspension at the age of 3 and 6 months was shorter than in control mice. These observations suggests that “young” PARP-1^−/−^ mice are more active and mobile, but they are physically weaker than PARP-1^+/+^ mice. With aging, their overactivity seems to decline faster as the differences between both groups disappeared.

### 3.6. Age-Related Dynamics of Biochemical Parameters

The dynamics of biochemical parameters in the serum of overnight fasting PARP-1^−/−^ and PARP-1^+/+^ mice is presented in Table 2. The level of total proteins was lower in PARP-1^−/−^ mice in comparison with PARP-1^+/+^ mice at the age of 4 months (*P* < .05) as well as at 20 months (*P* < .01), in part, due to a reduction in albumin level (*P* < .05). In the knock-out mice of both age groups, hypocalcaemia was also found (*P* < .01), perhaps due to albumin reduction leading to decrease in the level of the total calcium binding proteins. A decrease in serum levels of uric acid was observed in the old PARP-1^−/−^ mice as compared to PARP-1^+/+^ animals (*P* < .001). Four-month-old PARP-1^−/−^ mice showed a reduced activity of lactate dehydrogenase in the serum as compared with PARP-1^+/+^ mice of the same age (*P* < .05). In the 4-month-old knock-out mice, the level of *α*-amylase was also decreased compared to PARP-1^+/+^ mice (*P* < .002) although at the age of 20 months, the *α*-amylase level was not significantly different between both groups. There was a tendency toward the reduction in an activity of some other enzymes studied in PARP-1^−/−^ mice as compared to PARP-1^+/+^ ones, suggesting a general metabolism reduction in PARP-1 knock-out mice. There was no significant differences in glucose and lipid levels between PARP-1^−/−^ and PARP-1^+/+^ mice at young and old age (Table 2).

### 3.7. Survival and Life Span of Mice

The mean and maximum life span of mice in the PARP-1^−/−^ mice was reduced by 13.3% and 16.4%, respectively, as compared with these of PARP-1^+/+^ mice (*P* < .0002) (Table 3). According to the log-rank test [26], the difference in life span distributions between groups of PARP-1^+/+^ mice and PARP-1^−/−^ mice was significant with *P* < .00000108 (*χ* ^2^ = 23.8 on 1 degree of freedom). In the PARP-1^−/−^ group, mean life span of 10% most long-lived mice, were significantly shorter than that of wild type mice. The survival of PARP-1^−/−^ female mice was compared to the PARP-1^+/+^ female mice using the Gompertz model [27]. The conditional mortality and survival functions for this model are given by the formulas:

(1) $$\begin{array}{ll} \mu \left(x\right) & =\beta \;\;\exp\;\left(\alpha \left(x-x_{0}\right)\right),\\ S\left(x\right) & =\exp\;\left(\frac{\beta }{\alpha }\left(1-\exp\;\left(\alpha \left(x-x_{0}\right)\right)\right)\right). \end{array}$$

**Figure 7:**
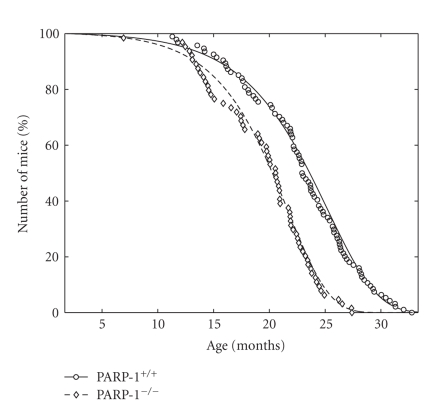
Empirical and approximated by Gompertz model survival curves of PARP-1^−/−^ and PARP-1^+/+^ mice. Surviving mice in each group were presented as percentage as a function of age.

Parameters *β* and *α* represent the initial level of mortality and the rate of aging of the population. The mortality rate doubling time (MRDT) is equal to log (2)/*α*. Estimated parameters of this model with confidence intervals are given in Table 3. While the initial levels of mortality for both groups of mice were the same, the aging rate of the PARP-1^−/−^ population was greater than PARP-1^+/+^ mice. The MRDT was shorter in the PARP-1^−/−^ mice as compared to the PARP-1^+/+^ group. The survival curve of PARP-1^−/−^ mice was significantly shifted to left (Figure 7) as compared to the survival curve of the PARP-1^+/+^ group.

### 3.8. Spontaneous Tumor Development

**Figure 8:**
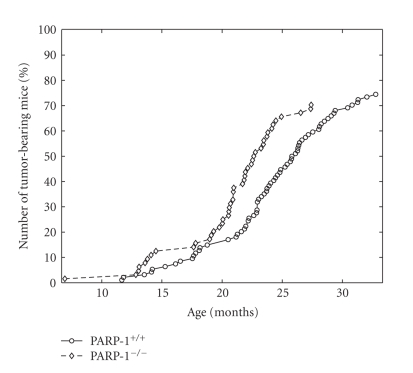
Total tumor yield curves for PARP-1^−/−^ and PARP-1^+/+^ mice. Number of tumor-bearing mice in each group were presented as percentage as a function of age.

At the autopsy, tumors were observed in 53 of 73 PARP-1^−/−^ mice (73%) and in 79 of 103 PARP-1^+/+^ animals (77%) (Table 3). However, the incidence of malignant tumors was higher by 20.5% in the knock-out mice compared to the wild type controls (*P* < .001). The difference in life span distributions between tumor-bearing female PARP-1^+/+^ mice and PARP-1^−/−^ female mice was significant with *P* < .00000842 (*χ* ^2^ = 19.8 on 1 degree of freedom). As shown in Table 3, the mean life spans of tumor-bearing PARP-1^−/−^ mice was significantly shorter than that in PARP-1^+/+^ mice. The mean life span of the most long living 10% of the knock-out survivors was also shorter than that of the control mice. The level of initial mortality (parameter *β* of the Gompertz model) was significantly greater for the group of wild type mice. In addition, the rate of population aging was higher and MRDT was lower in the PARP-1^−/−^ tumor-bearing mice as compared to the PARP-1^+/+^ group. The total tumor yield curve for PARP-1^−/−^ mice was significantly shifted to the left as compared to the curve of PARP-1^+/+^ mice (Figure 8).

The data on tumor sites, type, and latency in both groups are summarized in Table 4. The most frequent tumors were nonepithelial uterine tumors (mostly polymorphic sarcomas, as well as vascular tumors, namely, cavernous hemangiomas and hemangiendotheliomas) both in PARP-1^−/−^ and PARP-1^+/+^ mice. Epithelial uterine tumors (polyps and adenocarcinomas) were less common. Ovarian granulosa-theca cell tumors were frequently observed in PARP-1^+/+^ mice but not in PARP-1^−/−^ animals. Hemangiomas and hemangioendotheliomas have been found in other organs (liver, subcutaneous tissue). Moreover, both PARP-1^+/+^ and PARP-1^−/−^ mice developed epithelial tumors of the lung (adenomas, adenocarcinomas) and of the liver (hepatocellular carcinomas). In total, 120 cases of tumors in the PARP-1^+/+^ group, and 82 cases of tumors in PARP-1^−/−^ group were observed.

### 3.9. Mathematical Modeling of the Results

The Cox regression model [24] was used to estimate relative risk of death for female PARP-1^−/−^ mice compared to the PARP-1^+/+^ group:

(2) $$h\;(x,z)=h_{0}\;(x)\exp\;\left(z\beta \right),$$

where *h* (*t*, *z*) and *h*_0_ (*t*) denote the conditional hazard and baseline hazard rates, respectively, *β* is the unknown parameter for treatment group, and *z* takes values 0 and 1, being an indicator variable for two samples—the control (PARP-1^+/+^) and group of interest (PARP-1^−/−^). It can be seen that for all subgroups, the relative risk of death is higher for the PARP-1^−/−^ mice compared to PARP-1^+/+^ mice (Table 5). The highest risk was estimated for the fatal-tumor-free mice.

A semiparametric model of heterogeneous mortality [25] was used to compare PARP-1^−/−^ female mice to the group of the wild type female mice in terms of frailty distribution and baseline hazard. The PARP-1^+/+^ group was considered as the control. Survival and mortality rate functions are given by the formulas:

(3) $$\begin{array}{ll} & S_{\text{PARP}}\left(x\right)={\left(1+r\gamma \left(S_{\text{WT}}{\left(x\right)}^{-\sigma^{2}}-1\right)+r\gamma \sigma^{2}\frac{\alpha }{\beta }\left(\exp\;\left(\beta \left(x-x_{0}\right)\right)-1\right)\right)}^{-1/\gamma \sigma^{2}},\\ & \mu_{\text{PARP}}\;\left(x\right)=\frac{r\mu_{\text{WT}}\left(x\right)S_{\text{WT}}{\left(x\right)}^{-\sigma^{2}}+r\alpha \;\;\exp\;\left(\beta \left(x-x_{0}\right)\right)}{1+r\gamma \left(S_{\text{WT}}{\left(x\right)}^{-\sigma^{2}}-1\right)+\gamma r\sigma^{2}(\alpha /\beta )\left(\exp\;(\beta \left(x-x_{0})\right)-1\right)}. \end{array}$$

Parameter *σ* ^2^ indicates the presence of heterogeneity in the control population. Differences in the baseline hazard are controlled by parameters *α* and *β*. Parameter *α* reflects permanent (constant) decrease or increase of the baseline hazard compared to the control group, depending on whether *α* is greater or less than zero. Parameter *β* describes the amplification or disappearance of the *α*-effect, according to whether *β* is greater or less than zero. Differences in the frailty distribution are controlled by parameters *r* and *γ*. Parameter *r* < 1 shows an increase in the average robustness, while *r* > 1 indicates an accumulation of frail individuals in the population compared to the PARP-1^+/+^ group. Parameter *g* ≠ 1 shows an increase (*g* > 1) or decrease (*g* < 1) in the population heterogeneity.

To compare the survival function for PARP-1^−/−^ mice to the PARP-1^+/+^ group, three specifications of the model were considered. The first one deals only with the differences in the average frailties of the populations (*α* = 0, *r* ≠ 1, *γ* = 1). With the second specification, differences in the mean of the frailty distributions are accompanied by differences in the baseline hazards (*α* ≠ 0, *β* = 0, *r* ≠ 1, *γ* = 1). The third specification describes differences in survival patterns between the groups of interest and the control group as a combination of differences in the baseline hazard and both parameters of the frailty distribution (*α* ≠ 0, *β* ≠ 0, *r* ≠ 1, *γ* ≠ 1). Because these specifications are nested, the likelihood ratio statistics are used to determine which one gives the best fit to the data. The parameter estimates were obtained using maximum likelihood method, nonlinear optimization procedure [22], and a customized code in “MATLAB”; confidence intervals for the parameters were obtained using the bootstrap method [23]. The third specification of the model corresponds to the data better than the others (Table 6). The estimated parameter values of this specification are presented in Table 7. The PARP-1^+/+^ control group was heterogeneous (parameter *σ* ^2^ ≠ 0). The group of PARP-1^−/−^ mice had slightly increased baseline hazard compared to the control group (parameters *α* > 0, *β* > 0). The PARP-1^−/−^ group is frailer on average (*r* > 1) and more homogeneous (*γ* < 1), compared to the wild type mice.

## 4. Discussion

There is constant DNA damage caused by endogenous and exogenous sources and DNA lesions accumulate with age in individuals. The efficacy of DNA repair plays an important role in aging and in aging-related pathological changes, including cancer [28–33]. Recent studies using knock-out mice defective for various nucleotide excision repair genes revealed a crucial role of DNA repair as a longevity assurance pathway [8, 28]). PARP-1 is DNA nick senor and responds very rapidly to single-stranded DNA breaks. PARP-1 is functionally associated with several DNA repair pathways [8, 34]. An obvious question is whether the downregulation of PARP-1 or PAR formation would be a causal factor for aging phenotype or affecting longevity.

In the current study, we investigated the role of PARP-1 in aging and used PARP-1^+/+^ and PARP-1^−/−^ female mice of the 129/Sv background and monitored them for the period over 24 months. The reason to use females is that female mice are less aggressive than males and can be grouped as a larger cohort to make more powerful statistical comparisons, and, moreover, we can monitor estrous cycles, a reliable noninvasive parameter of aging [35]. Interestingly, we found that the mean life span of all PARP-1^−/−^ mice and also the mean life span of the last 10% survivors were significantly reduced in comparison with the respective wild type control groups (Table 3). The same tendency was also observed during the analysis of the maximum life span and median life span. The aging rate was significantly increased and the mortality rate doubling time was shortened in PARP-1^−/−^ mice as compared with PARP-1^+/+^ mice. In addition, monitoring of estrous function in the PARP-1^−/−^ mice revealed an accelerated aging of the reproductive function as compared to PARP-1^+/+^ mice. As summarized in Table 8, PARP-1 deletion accelerated aging, as is revealed by many age-related changes, such as body weight and temperature, locomotor activity, and physical strength. These data indicate that PARP-1 deficiency or low PAR formation leads to acceleration of aging, reduction of life span, and early onset of tumor development. These results are in good agreement with the observations on obesity and increased metabolism rate in the PARP-1 knock-out mice [18]. Whereas aged PARP-1^+/+^ mice showed an increase of the serum level of the uric acid that has antioxidant properties [36, 37], which might reflect an antiaging mechanism, PARP-1 knock-out mice did not show age-related increase in the level of uric acid. Instead, the uric acid level was two-fold less than that in the wild type mice at the age of 20 months. Finally, these data are in agreement with the study of Grube and Bürkle [14] who described a positive correlation between the poly(ADP-ribosyl)ation capacity of mononuclear blood cells with longevity of mammalian species and also with additional correlation studies showing that PARP activity is decreased in aging human samples [16, 17]). This conclusion is also consistent with the notion that the efficient DNA repair, proper poly(ADP-ribosyl)ation, and the life span are positive, correlated during aging (See reviews by Bürkle et al. [38, 39]; Hoeijmakers [33]).

In addition to the change of aging-related physiological parameters, tumor development is a pathological process that is also associated with aging. While it could be caused by a deficiency DNA repair and genomic instability, other molecular pathways including cell cycle control, gene transcription, and environmental exposure also play an important role. The major tumor types observed in the present study, such as tumors in the uterus, mammary gland, lung, and liver have been reported in PARP-1 deficient mice in previous studies using recombinant genetic mutants. PARP-1 knock-out mice do not often develop spontaneous tumors, whereas in DNA repair deficient background, PARP-1 knock-out mice develop a high frequency of tumors. For example, in the Ku80^+/−^ background, a high frequency of liver cancer was observed [40], whereas in p53 heterozygous and homozygous background, PARP-1 null mice develop a variety of tumors, including mammary gland carcinoma, lung cancer, as well as brain tumors (see [2, 41]). The high frequency of uterine neoplasm in PARP-1^−/−^ female mice is reminiscent of a previous report correlating the loss of PARP-1 and development of endometrial carcinomas in humans [42].

In summary, our study using a genetically engineered animal model has demonstrated that knock-out of PARP-1 leads to acceleration of aging, reduction of life span, as well as to earlier and more aggressive tumor development, indicating that DNA repair molecule PARP-1 and its role in metabolism of poly(ADP-ribose)ation positively contribute to the longevity.

## Figures and Tables

**Table 1:** Age-related dynamics of estrous functional parameters in PARP-1^−/−^ and PARP-1^+/+^ mice.

Age (months)	Number of mice	Length of estrous cycles (days)	Rate (%) of			Number of mice with irregular cycles (%)
			<5 days	5–7 days	>7 days	
PARP-1^+/+^						

2	37	6.7 ± 0.34	14	52	34	18
5	27	5.55 ± 0.50^a^	41	36	23	22
8	31	6.08 ± 0.42^b^	33	42	25	35
14	18	7.30 ± 1.03	20	50	30	50
17	39	7.42 ± 0.41	13	45	42	23
20	27	6.1 ± 0.35	19	65	16	30

PARP-1^−/−^						

2	34	7.4 ± 0.49	5	59	36	13
5	23	6.64 ± 0.45^a^	23	50	27	17
8	22	6.59 ± 0.56^a^	32	36	32	27
14	18	7.91 ± 0.86	27	18	55	44^b^
17	27	8.72 ± 0.50*	17	28	55	11
20	18	5.7 ± 0.56	14	72	14	72**

Significant in comparison with control: *P < .05, ** −*P* < .001. The difference from the parameter at the age of 17 months in the same group: a: *P* < .01, b: *P* < .05.

**Table 2:** Age-related dynamics of biochemical parameters in the serum of PARP-1^−/−^ and PARP-1^+/+^ mice.

Parameters	PARP-1^+/+^		PARP-1^−/−^	
	4 months, *n* = 10	20 months, *n* = 10	4 months, *n* = 10	20 months, *n* = 10
Total protein, g/l	45.19 ± 1.15	48.33 ± 1.10	41.88 ± 1.47^a^	25.22 ± 0.46^c,*##*^
Albumin, g/l	21.69 ± 0.52	22.04 ± 0.42	19.18 ± 1.13*	20.99 ± 0.29^*#*^
Glucose, mM/l	7.27 ± 0.30	6.89 ± 1.33	7.51 ± 0.60	8.31 ± 1.57
Cholesterol, mM/l	3.09 ± 0.09	2.86 ± 0.08	2.86 ± 0.17	2.83 ± 0.15
Triglycerides, mM/l	0.89 ± 0.04	0.88 ± 0.08	0.75 ± 0.07	1.00 ± 0.29
Urea, mM/l	10.46 ± 0.43	9.33 ± 0.27	8.79 ± 0.94	10.31 ± 0.41
Creatinine, *μ*M/l	31.70 ± 1.34	32.17 ± 3.81	27.83 ± 1.68	31.45 ± 6.0
Uric acid, *μ*M/l	43.60 ± 5.46	133.92 ± 8.94^c^	54.10 ± 21.55	66.27 ± 8.10^*###*^
Calcium, mM/l	1.87 ± 0.05	2.09 ± 0.03^b^	1.43 ± 0.16**	1.98 ± 0.04^b*#*^
Alanine-amino-transferase. U/l	56.50 ± 4.85	43.42 ± 2.48^a^	50.82 ± 4.48	44.73 ± 5.62
Aspartate-amino-transferase, U/l	341.80 ± 40.62	231.25 ± 21.86^a^	259.33 ± 25.93	209.36 ± 16.38
Alkaline phosphatase, U/l	122.80 ± 10.21	129.83 ± 17.17	125.67 ± 8.03	92.00 ± 10.76^a^
Lactate dehydrogenase, U/l	1330 ± 140.93	897.42 ± 83.54^b^	917.91 ± 131.33*	780.64 ± 44.0
*γ*- Glutamyl-transferase, U/l	6.38 ± 1.92	4.55 ± 0.29	4.9 ± 0.40	5.38 ± 0.6
*α*-Amylase, U/l	1518.33 ± 59.93	2519.17 ± 336.99^b^	542.00 ± 186.38***	2802.0 ± 562.86^b^

Significant in comparison with the control of the same strain at the age of 4 months: a: *P* < .05; b: *P* < .01; c: *P* < .001; Significant in comparison with the PARP-1^+/+^ at the age of 4 months: *: *P* < .05; **: *P* < .01; ***: *P* < .002. Significant in comparison with the PARP-1^+/+^ at the age of 20 months: ^*#*^: *P* < .05; ^*##*^: *P* < .01; ^*###*^: *P* < .001.

**Table 3:** Parameters of survival, life span and tumorigenesis in PARP-1^−/−^ and PARP-1^+/+^ mice.

Parameters	PARP-1^+/+^	PARP-1^−/−^
Number of mice	103	73
Mean life span (days, mean ± S.E.M.)	678 ± 14.2	588 ± 14.4**
Median (days)	686	597
Mean life span of last 10% of survivors (days)	919 ± 11.6	778 ± 14.3**
Maximum life span (days)	983	822
Aging rate, *α* (days^−1^)	0.00771 (0.00760; 0.00782)	0.00932 (0.00926: 0.00956)*
MRDT (days)	89.88 (88.6; 91.22)	74.36 (72.53; 74.89)*
Number of tumor-bearing mice	79 (76.7%)	53 (72.6%)
Number of malignant tumor-bearing mice	48 (46.6%)	49 (67.1%)***
Mean life span of tumor-bearing mice (days, mean ± S.E.M.)	706 ± 17.6	612 ± 19.2**
Total number of tumors	120	82
Total number of malignant tumors	59 (49.2%)	59 (72.0%)*

*α* in Gompertz model: *R* = *R*_0_ (exp)*αt*, where *R*_0_ = mortality rate in the time of *t* = 0; 95% confidence limits are given in parentheses; MRDT, mortality rate doubling time.

The difference with the PARP-1^+/+^ is significant: *: *P* < .05; **: *P* < .002; ***: *P* < .001.

**Table 4:** Tumor site and type in PARP-1^−/−^ and PARP-1^+/+^ mice.

Tumor localization and type		PARP-1^+/+^		PARP-1^−/−^	
		Number of tumor-bearing mice (%)	Survival, days	Number of tumor-bearing mice (%)	Survival, days
Uterus	Sarcoma	37 (36%)	713 ± 21.0	28 (38%)	659 ± 22.9
	Adenocarcinoma	5 (5%)	799 ± 41.2	5 (7%)	709 ± 33.5
	Hemangioma	19 (18%)	761 ± 29.9	1(1%)***	679
	Hemangioendothelioma	3 (3%)	635	2 (3%)	664
	Polyp	8 (8%)	718 ± 65.6	2 (3%)	601
Ovary	Adenocarcinoma	1 (1%)	961	—	
	Granulesa-theca cell tumor	12 (12%)	692 ± 52.0	1 (1%)**	734
	Hemangioma	5 (5%)	798 ± 71.8	6 (8%)	668 ± 55.1
	Cystadenoma	2 (2%)	878 ± 43.8	1 (1%)	534
Mammary gland	Adenocarcinoma	3 (3%)	748 ± 163.4	4 (6%)	480 ± 26.9
Lung	Adenocarcinoma	4 (4%)	596 ± 54.8	7 (10%)	480 ± 26.9
	Adenoma	8 (8%)	702 ± 72.5	4 (6%)	749 ± 12.7
Liver	Hepatocellular carcinoma	3 (3%)	810 ± 20.6	6 (8%)	664 ± 21.4***
	Hemangioendothelioma	—		2 (3%)	630 ± 35.6
	Haemangioma	3 (3%)	923 ± 53.8	2 (3%)	628
Haematopoietic system	Malignant lymphoma	5 (5%)	711 ± 37.1	6 (8%)	593 ± 40.1*
	Thymoma	—	—	1 (1%)	208
Soft tissue	Subcutaneous angiosarcoma	1 (1%)	788	—	
	Hemangioendothelioma	1 (1%)	414	2 (3%)	497 ± 103.1
Skin	Squamous-cell carcinoma	—	—	1 (1%)	392
Colon	Adenocarcinoma	—		1 (1%)	393

Significant in comparison with the PARP-1^+/+^: *: *P* < .05; **: *P* < .01; ***: *P* < .001.

**Table 5:** The Cox's model parameters for different subgroups of PARP-1^+/+^ and PARP-1^−/−^ mice.

Hazard PARP-1^−/−^ versus PARP-1^+/+^	B	exp (*β*)	se (*β*)	p
All mice	0.83	2.3	0.18	2.10e-06
Mice with tumors	0.91	2.47	0.21	1.50e-05
Mice with fatal tumors	0.93	2.54	0.24	9.40e-05
Mice with nonfatal tumors	0.97	2.65	0.27	3.90e-04

**Table 6:** Comparison of three models using the likelihood ratio method.

	*r*, *σ*2	*α*, *r*, *σ*2	*α*, *β*, *r*, *γ*, *σ* ^2^
−LogLik	1026.77	1021.48	1009.32
*P*	5.710213184310087e-04	2.28817665292e-03	—*

* The estimated parameter values of this specification are presented in Table 7.

**Table 7:** Estimated values of parameters with confidence intervals.

Parameter	*α*	B	R	Γ	*σ* ^2^
Value	2.33e-06	9.92e-06	1.85	0.17	0.99
CI	(2.31e-06; 2.36e-06)	(9.87e-06; 9.94e-06)	(1.81; 1.87)	(0.16; 0.19)	(0.97; 1.03)

**Table 8:** Effects of PARP-1 knockout in female mice.

Parameter	PARP-1^−/−^ versus PARP-1^+/+^	Comment
Body weight	Increased after the age of 21 months	Accelerated aging
Food consumption	No difference	No effect
Body temperature	Increased	Accelerated aging
Maturation (vagina opening)	accelerated	Accelerated maturation
Estrous function	Rate of mice with irregular cycles increased	Accelerated aging

*Locomotor activity*		Young −/− mice more active, but more physically weak than +/+ mice; accelerated aging
No. of crossed squares	Increased	
No. of vertical racks	Increased	
Duration of standing reaction	Decreased	
Duration of the 1st and 2nd suspension time	Decreased	

*Biochemical parameters*		
Total protein	Decreased with age	Accelerated aging
Uric acid	Decreased at the age of 20 months	
Calcium level	Decreased at the age of 4 months	Prone to osteoporosis
Alanine aminotransferase	No age-related decrease	Disturbance in the age pattern
Lactate dehydrogenase	No age-related decrease	
*α*-amylase	Decreased at the age of 4 months	Accelerated aging
Mean life span	Reduced	Accelerated aging
Mean life span of last 10% of survivors	Reduced	
Maximum life span	Reduced	
Aging rate	Increased	
MRDT	Reduced	
No. of malignant tumor-bearing mice	Increased	Progression of carcinogenesis
Total number of malignant tumors	Increased	
